# Miscellaneous

**Published:** 1896-05

**Authors:** 


### SELECTIONS AND ABSTRACTS.

### MISCELLANEOUS.

### A Physician and Surgeon's Right to Compensation.

A physician and surgeon's right to compensation depends upon contract, express or implied. The services of a physician being valuable, the law will imply a contract to pay a reasonable consideration therefor by any one receiving the benefit of such services. As example and authority it has held that where a physician was summoned to attend his aunt upon matters of business, but while with her rendered valuable medical attention which she accepted. The court said he was entitled to recover for such services.

A demand by a physician against the estate of a decedent must be proved strictly; no promise can be implied in favor of claimant without undoubted proof of service rendered. In Indiana it has recently been held that a demand by a physician against the estate of a decedent must not only be proved strictly, but claimant must also allege and prove that he was at the time the services were rendered a regularly qualified and licensed physician.

It has been held, that where a physician is called in consultation by an attending physician, he may recover his fee from the patient, although there may have been an engagement between the attending physician and the patient that the former should pay the consultation fee.

An implied contract is in the first instance usually with the patient, or where one stands in such relation with the patient as to be liable for necessaries furnished him, as the relation of parent and child, husband and wife, guardian and ward, the implied promise is by such person.

In case of surgeons, where patients suffering from dangerous diseases are left in their care, operations may be performed on them without notice to those who placed them in the surgeon's care. And if death results the surgeon may recover against them for compensation, because such performance or operation is within the scope of a surgeon's authority and judgment. It is not necessary or incumbent on the surgeon to prove that it was necessary or proper under the circumstances; and a defendant cannot escape payment of such fee on the ground that the surgeon gave him no notice, or prove that it would have been dangerous to the patient or patients to wait until notice could be given.

A promise of a third person to pay for medical services rendered another may be inferred as in any other case, where the circumstances are strong enough. Thus, where a person called at the office of a physician and left his business card, having written on it, "Call on Mrs. A., No. --St.," with the clerk, requesting him to tell the physician to call at once, it was held that he became liable to pay for the physician's attendance upon Mrs. A.

Where a justice of the peace acting as coroner requested a physician to examine a body over which an inquest was being held, it was decided that he was entitled to an allowance from the county.

In an action to set aside a will, two physicians were appointed by the court to examine and report whether the widow of the testator was pregnant. *Held*, that they were *quasi* officers of the court, and were entitled to be paid out of the estate, without awaiting the termination or event of the litigation.

A county is not liable to a physician for services rendered to paupers. There is no implied promise by a county to pay for services rendered by a physician or surgeon to the poor if there has been no judicial ascertainment that the person cared for is a pauper. The presumption is rather that the physician looks to his patient for pay or bestows his services in charity.

As a rule the majority of the States operate under a statute providing for a contract with physicians to attend the poor in certain districts. But in some cases a county has been held answerable to physicians for services rendered in cases of emergency to persons who are chargeable upon the county or who became so immediately afterward. So held in Penn., Kas. and Indiana.

It is no part of the duty of a physician employed under contract by a county to treat its poor to make a *post mortem* examination of the body of a dead pauper, and when he does so at the request of the coroner he is entitled to compensation.

*No recovery can be had where there is no implied promise to pay.* Thus, where a person, or messenger, was sent for a physician, and not finding the one sent for, spoke to another, and on his arrival, before any service was performed, in his presence, the manner of his employment, and the value of the service to be performed were explained to the patient. *Held,* in an action by the physician against the messenger, that the latter was not liable.

Where a physician was employed to attend the employees of a plantation, found a surgical operation necessary on one of the employees and requested the overseer to send for another physician, who came and performed the operation without any assistance from the plantation physician. *Held,* that no action could be maintained against the plantation physician by his brother physician for the services rendered.

A physician cannot recover of a brother of an insane person for medical attendance rendered the latter, at the request of such brother, unless there was an employment under such circumstances as to show an intention on the part of such brother to pay for the services, and so understood by him and the physician.

Where a party was under bond for the support of a patient, who had called in a physician without the consent of the defendant bondsman, no implication arises that the defendant will be answerable for the physician's services.

If a physician or physicians attending a woman deem it necessary, for the preservation and prolongation of her life, to perform an operation, they are justified in doing so, if she consents, whether her husband consents or not.

The degree of care and skill required of physicians is that reasonable degree of care and skill which physicians ordinarily exercise in the treatment of their patients.

A physician does not insure that his treatment will be successful, but he is bound to bestow such reasonable ordinary care, skill, and diligence as physicians in the same neighborhood, in the same general line of practice, ordinarily have and exercise in like cases.

One who holds himself out as a healer of diseases, and accepts employment as such, must be held to the duty of reasonable skill in the exercise of his vocation; failing in this, he must be held liable for any damages proximately caused by unskilled treatment of his patient.

The employment of a physician continues while sickness lasts, unless put an end to by the assent of the parties or revoked by the express dismissal of the physician.

The mere failure to effect a cure does not even raise a presumption of a want of proper care, skill, and diligence in a physician.

A clairvoyant physician is liable for failure to exercise the ordinary skill and knowledge of a physician in good standing, practicing in the vicinity, and not merely the ordinary skill and knowledge of clairvoyant. If he holds himself out as a medical expert, and accepts employment as a healer of disease, but relies for diagnosis and remedies upon some occult influence exerted upon him, or some mental intuition received by him when in an abnormal condition, he takes the risk of the quality or accuracy of such influence or intuition.

It is the duty of a surgeon who has set a broken leg to give proper instructions for the use and care of it; and for failure to do so he is liable in case of a resulting injury.

A physician employed by a city or county to treat patients in a jail or in an almshouse, will not be relieved from liability to such patient therein for failure to exercise ordinary care and skill, although he is not paid by the patient.

Where a physician or surgeon is employed to treat a patient, without any express special contract defining the character and extent of his duty and undertaking, the form of remedy is either an action of assumpsit or case may be maintained for the breach of the implied obligation arising from such employment, caused by unskillful, negligent, and improper treatment of the patient.—By MR. R. D. FISHER, Reporter Supreme Court of Indiana, *Fort Wayne Medical Magazine.*

---

### PRESENT STATUS OF CEREBRAL SURGERY.

At the twenty-fourth session of the German Congress of Surgery, held at Berlin, Professor von Bergmann said: Thanks to the introduction of antiseptic methods and the steady advance realized by physiologists and clinicians in the domain of cerebral localization, surgeons now unhesitatingly, and frequently with success, undertake t. e treatment of diseases, which have hitherto been regarded as incurable. Unfortunately, for various reasons, and particularly the difficulty or impossibility of arriving at a correct diagnosis in many of the cases, the field of cerebral surgery will always be limited. Thus, with regard to tumors of the brain, statistics show that operation is practicable only in 29 per cent. of all cases, and that three-fourths of these cannot be operated upon, on account of the impossibility of arriving at a diagnosis. All our efforts should, therefore, be directed, on one hand, toward defining the diagnostic indications, and, on the other hand, toward perfecting more and more our operative methods. Considerable advance has already been made in the latter direction by the introduction of extensive osteoplastic resections of the skull, which render possible the extirpation of large tumors. Nothing is of more importance than speed in operation, and this can be obtained by the use of an instrument which I am enabled to show you, consisting of a circular saw, set in extremely rapid motion by means of a small electric motor. With this instrument a large bone flap may be sawn out in a few moments, the operation being completed by the aid of a sharp chisel, in order to avoid, as far as possible, injuring the dura-mater.

Numerous attempts have been made to cure epilepsy by surgical treatment; but these attempts have proved unsuccessful, except in those forms of epilepsy, which are due to a lesion of the cortical motor centers. It is necessary, however, to be guarded in drawing conclusions from the results obtained, as cases of temporary recovery of epileptic patients, consequent upon simple trephining or excision of a cicatrix of the scalp, are of frequent occurrence. For my own part, I have seen epileptic attacks cease during the time necessary for the healing of the wound in an individual who had undergone amputation of the leg. It is impossible to approve of operations, which consist in extirpating, in epileptic patients, portions of the cerebral cortex which are apparently healthy, but which are considered to be the starting point of epileptic seizures. Extirpation, indeed, is only justified in cases in which a distinct lesion, a cyst, for example, exists.

Great success has been obtained in operations for abscesses of the brain. Surgeons have even had the courage to attack thrombosis of the sinuses and suppurating leptomeningitis. In this case also the diagnosis is far from being always possible, and in many instances of unsuspected abscesses intervention is, therefore, abstained from. In the very short time at my disposal I cannot deal as fully with the treatment of intracranial abscesses as would be desirable in view of the importance of the subject. I shall, therefore, confine myself to briefly considering the most frequent form of these abscesses, viz., those which follow a suppurating otitis media. In one-fourth of such cases, the cause of the suppuration is a cholesteatoma of the middle ear. Intracranial abscesses, developing under such conditions by continuity, are situated either outside, or within, the dura-mater, or in the interior of the cerebral substance, most frequently in the temporal lobe. In order to reach these abscesses, which are comparatively accessible and, as a rule, located in the portion of the cranial cavity situated above the tympanum, I have devised a method of operation, which, in my opinion, is preferable to the simple trephining usually resorted to in such cases. This procedure consists in resecting a quadrangular portion of the wall of the skull immediately above the external auditory meatus. With this object in view, a cutaneo-musculo-periosteal flap, left adherent at its upper part, is made by three incisions, one horizontal, beginning above the zygoma and ending behind the mastoid process, the others vertical and perpendicular to the former, one in front of the tragus, the other behind the mastoid process, both being carried upward to the height of about four centimeters. Removal of the bone beneath this flap gives unobstructed access to the interior of the skull, and greatly facilitates the task of the operator.

In thrombosis of the sinuses, surgical intervention has given unexpected results. The object of this intervention, as is well known, is to reach directly the transverse sinus—which is the one usually involved—through the mastoid process, the transverse sinus being situated beneath the middle portion of the latter. It is unnecessary to insist upon the details of this operation: incision of the sinus after exploratory puncture, evacuation of the clots, disinfection and plugging, and, lastly, ligature of the internal jugular vein. Of thirteen cases operated upon, recovery followed in six. Death is usually due either to pyemia or to suppurating lepto-meningitis.

Of late, operations have been undertaken, in order to remedy the inconveniences resulting from exaggerated intracranial pressure, from whatever cause. In this connection Quincke's attempts at tapping the lumbar portion of the spinal cord, in cases of basilar meningitis, are worthy of mention. The results obtained so far have only amounted to some improvement of short duration. In no instance has a fatal termination been averted by such puncture; but, on the other hand, it has proven useful as a diagnostic measure, because, if bacteriological examination of the liquid withdrawn reveals the presence of Koch's bacillus, it is evident that it is a case of tubercular meningitis. Possibly puncture may also be of value as an aid to diagnosis, when the existing symptoms do not permit of a differential diagnosis between abscess of the brain and more or less localized suppurating meningitis.—*Practitioner.*

---

HYDROZONE IN PURULENT OTITIS MEDIA.—A REPORT OF A CASE SUPPOSED TO INVOLVE INFLAMMATION OF THE MASTOID.

Dr. Wm. Clarence Boteler, of Kansas City, Mo., in the *Medical Bulletin*, Philadelphia, Pa., February, 1896, says:

"On November 4th, 1895, I was consulted at my office by Robert P——, aged twenty-four years; occupation, laborer in the Armour Packing Company. The patient complained that for about four weeks he had been suffering from intense pain in his left ear, making it impossible for him to sleep at night, or rest during the day. The pain was so severe that at times he apparently lost consciousness and it seemed to extend through his entire brain. Upon inspection, the man's face was found terribly deformed; an edematous swelling the size of one half of an ordinary loaf of baker's bread occupied the usual location of the ear and the surrounding muscles. The auricle of the ear was almost buried in edematous tissue; upon palpation, the part was found intensely tender, and deep pressure evoked expressions of excruciating pain. The integument and subcutaneous tissue were thoroughly infiltrated. Ichorous, fetid pus was slowly exuding from an almost imperceptible meatus. The patient expressed feelings of chilliness, showing a possible septic contamination of his system. Every indication and sign pointed to possible suppuration of the mastoid cells—tenderness upon pressure over the mastoid being very marked. Efforts to localize the tenderness, whether in external meatus or mastoid, for discriminating diagnosis, were unsatisfactory. I concluded to withhold a positive diagnosis as to whether the condition was purulent otitis media or suppurative inflammation of the mastoid, and used tentative treatment for a short while. I immediately placed the patient under heroic doses of elixir of the six iodides internally. After laborious effort I succeeded in separating the edematous tissue sufficiently to admit the introduction of a small Eustachian catheter into the external meatus. Through this, with a small hard rubber syringe, I injected four times daily about one-half an ounce of hydrozone, allowing it later to drain away, advising hot fomentations. The patient was confined to his bed and the best possible hygienic surroundings provided. In twenty-four hours after the treatment was commenced, the intensity of the odor, amount and character of the discharge had manifestly lessened, the swelling was reducing and the patient feeling better. The edema being lessened, the aperture was enlarged. I now recommended the injection of hydrozone through a catheter of larger caliber, every hour, requiring the head to be kept turned to the opposite side for ten minutes to allow the percolation of the hydrozone as deeply as possible into the middle ear, before reversing the position to allow drainage. We continued this treatment for a week, the man's recovery progressing with remarkable rapidity, his pain and the constitutional symptoms having disappeared about the third day. At the end of eight days the swelling had entirely disappeared, his features were again normal, and he expressed himself as perfectly well. An examination showed a circular perforation in the ear drum the size of a shot, proving that the case had been one of purulent otitis media, with septic contamination of the patient's system, and infiltration of the surrounding cutaneous tissues. Small incisions were made at two different places to permit the exit of pus from the integument. The mastoid was found not involved. The rapidity with which the disease yielded after the introduction of hydrozone through the catheter into the middle ear impressed me with the wonderful value of the preparation ; for, struggling with such cases during a practice of seventeen years, I have never seen its efficiency equalled by any medicinal or operative procedures."

